# Sex difference in age-related changes in knee extensor strength and power production during a 10-times-repeated sit-to-stand task in Japanese elderly

**DOI:** 10.1186/s40101-015-0072-4

**Published:** 2015-11-14

**Authors:** Naoko Yanagawa, Teruichi Shimomitsu, Masashi Kawanishi, Tetsuo Fukunaga, Hiroaki Kanehisa

**Affiliations:** Japan Health Promotion and Fitness Foundation, 2-6-10 Higashishinbashi, Minato-ku, Tokyo 105-0021 Japan; National Institute of Fitness and Sports in Kanoya, 1 Shiromizu, Kanoya, Kagoshima 891-2393 Japan

**Keywords:** Field test, Functional power, Strength capability

## Abstract

**Background:**

For middle-aged and elderly women, age-related decline in an index representing power production during STS task (STS-PI), calculated by using an equation reported previously, has been shown to be greater than that in the force generation capability of lower extremity. Whether this is specific to women remains unclear. This study examined how the age-related changes in knee extensor strength and power production during STS differ between Japanese men and women aged 65 years or older.

**Methods:**

The time taken for a 10-times-repeated STS test (STS time) and force developed during maximal voluntary isometric knee extension (KE-F) were determined in Japanese younger-old (262 men and 285 women) aged 65–74 years and older-old (96 men and 89 women) aged 75–90 years. STS-PI was calculated using the following equation: STS-PI = (body height − 0.4) × body mass × 10/STS time.

**Results:**

KE-F and STS-PI were significantly greater in the younger-old than in the older-old group (*p* < 0.0001) and in men than in women (*p* < 0.0001). STS-PI and KE-F, expressed as the percentages of the mean value of the corresponding variable for the younger-old group (%STS-PI and %KE-F, respectively), were negatively correlated to chronological age in both men (*r* = −0.386 and *r* = −0.269, respectively, *p* < 0.0001) and women (*r* = −0.504 and *r* = −0.294, respectively, *p* < 0.0001). Regression slopes in the relationship between age and %KE-F were not significantly different between men (−1.521) and women (−1.618). However, regression slope in the relationship between age and %STS-PI was significantly steeper in women (−3.108) than in men (−2.170) (*p* < 0.05). In OOG, %KE-F had no significant effect of sex, but %STS-PI was significantly lower in women than in men (*p* < 0.001).

**Conclusions:**

In Japanese men and women aged 65 years or over, age-related loss in power production during STS is steeper in women than in men, with greater magnitude than that in knee extensor strength. This suggests a higher priority of improving power generation capability during whole-body movement such as STS in older women than in older men.

## Background

Sit-to-stand (STS) test is extensively used as one of the representative tests examining physical functions. It is a whole-body movement without support, employs a motor pattern commonly used in daily living, and requires the performer to adjust body balance continuously [1]. Thus, the STS test is a good candidate to reveal functional limitations of the neuro-musculo-skeletal system [2].

For the elderly, although various factors such as sensation, speed, balance, and psychological status in addition to strength influence the time required to perform a given number of repetitions in the STS task, knee extensor strength is the most important variable for explaining the variance in it [3]. However, the sex difference in STS time cannot always reflect that in knee extensor strength [4, 5]. As an approach to this issue, Takai et al. [5] expressed 10-times-repeated STS performance as a power index (STS-PI) by using an equation involving leg length, body mass, height of chair, acceleration of gravity, and time taken for the task as variables and examined its associations with the cross-sectional area of the quadriceps femoris muscle and knee extension force in elderly individuals. In terms of the results, STS-PI was highly correlated to the two variables, but the corresponding associations for STS time were not significant. The power values calculated using an equation with body mass and STS performance as variables have been shown to be strongly correlated to the average and peak power values developed in the STS task, derived from force-platform and high-speed motion analyses [1]. These findings indicate that the STS-PI is a valid indicator for conveniently assessing the force and/or power generation capability of lower-body muscle. At the same time, we can say that, if one intends to examine age-related or sex-related difference in STS performance in relation to force generation capability, the STS performance should be expressed as a power value.

A prior study [6] that examined Japanese women aged 50 years or more reported that age-related decline in the STS-PI calculated by the equation reported by Takai et al. [5] was greater than those in the lean tissue mass of lower extremity and knee extension torque. This suggests that, for older women, the age-related loss in the power generation capability in whole-body movement such as STS is greater than that in the force generation capability of the prime movers. However, whether this is specific to women is unclear. From the findings of Petrella et al. [7], peak power during a STS task was 110 % higher in older men than in older women, whereas the sex difference in young adults was only 22 %. The execution of STS task involves muscle strength development of the lower body and wide ranges of joint motion, associated with an effort for controlling body balance [2]. Thus, not only the level of muscle strength but also the coordination of strength exertion influence on STS transfer [8]. A retrospective analysis of top age-group weightlifting and powerlifting records has indicated that the rates of age-related declines in performances were greater in women than in men in the whole-body movement tasks that require greater involvement of explosive power and complex action [9]. Considering these aspects, it can be assumed that the age-related loss in the power generation capability in whole-body movement such as STS may be greater in older women than in older men. In any case, no studies have examined how age-related loss in power development during the STS task differs between elderly men and women, in relation to that in the force generation capability of the prime movers. Elucidation of this is essential to discuss the importance of improving the power generation capability in whole-body movement as a strategy of physical training for elderly women because of the close association between the power generation capability of lower extremity and the performances of activities of daily living [10, 11] and lower functional mobility in older women than in older men [12].

The present study determined the time taken for a 10-times-repeated STS test (STS time) and force developed during maximal voluntary isometric knee extension (KE-F) in Japanese men and women aged 65–90 years. STS-PI was calculated using body height, height of chair, body mass, and STS time as variables. The purpose of this study was to elucidate the sex-related differences in the age-related changes in knee extensor strength and STS-PI in the elderly. We hypothesized that, as compared to older men, older women would show a steeper age-related loss in STS-PI, with greater magnitude than that in knee extensor strength.

## Methods

### Subjects

A total of 732 Japanese men (*n* = 358) and women (*n* = 374) aged 65–90 years voluntarily participated in this study. In this study, the collection of data was conducted from July to December in 2011 and 2012. In all experimental days, the measurements were performed from 9:00 to 12:00. The results of two-way analysis of variance (ANOVA) with a Scheffe test indicated that each of age and the measured variables had no significant effect of time (2011 vs. 2012), without significant interaction with sex. All participants answered to a written questionnaire, which was made in accordance with American College of Sports Medicine guidelines for exercise testing and prescription [13], asking about their background characteristics and health status. None was unable to go out unsupported or refrained from going out alone because of decreased physical capacity. In addition, no subjects had a history or evidence of lower extremity pain, unstable cardiovascular disease, or other medical condition that would be contraindicated for performing strength testing of the lower extremity and a 10-times-repeated STS. In addition, their participations in sports or physical exercises during daily living were assessed with lifestyle questionnaire. Among the subjects, 67 % of all men and 57 % of all women participated in a physical exercise such as walking, jogging, cycling, aerobic dance, aquabics, yoga, swimming, Tai Chi Chuon, ground golf, or social dance, at least once a week. The main type of the physical exercise was walking (46 % for men and 32 % for women). The subjects were allocated to the younger-old (262 men and 285 women aged 65–74 years, YOG) or the older-old (96 men and 89 women aged 75–90 years, OOG) group. Mean ± standard deviation (SD) of age for each group was 69.7 ± 2.8 years for men and 69.0 ± 2.7 years for women in YOG and 78.4 ± 3.4 years for men and 79.0 ± 3.2 years for women in OOG. Physical characteristics of each subject group are summarized in Table 1. This study was approved by the Ethics Committee of the National Institute of Fitness and Sports in Kanoya and was consistent with the institutional ethical requirements for human experimentation in accordance with the Declaration of Helsinki. The subjects were fully informed of the purpose and risks of the experiment and gave their written informed consent.

**Table 1:** Physical characteristics of the subjects

				Two-way ANOVA		
Variables	Sex	YOG	OOG	Age	Sex	Age × sex
Body height, m	M	1.636 ± 0.057^ab^	1.608 ± 0.062^b^	*p* < 0.0001	*p* < 0.0001	*p* = 0.215
	W	1.521 ± 0.050^a^	1.482 ± 0.053			
Body mass, kg	M	62.6 ± 7.7^ab^	59.5 ± 8.3^b^	*p* < 0.0001	*p* < 0.0001	*p* = 0.845
	W	53.5 ± 7.4^a^	50.7 ± 7.2			
BMI	M	23.39 ± 2.60	23.00 ± 2.69	*p* = 0.399	*p* = 0.802	*p* = 0.475
	W	23.14 ± 3.21	23.11 ± 3.21			

Values are means ± SDs

*M* men, *W* women, *YOG* younger-old group, *OOG* older-old group

^a^Significantly different from OOG at *p* < 0.0001 as a result of Scheffe test

^b^Significantly different from women at *p* < 0.0001 as a result of Scheffe test

### Measurements

#### Anthropometry

Height and body mass were measured using standard techniques to the nearest 0.1 cm and 0.1 kg, respectively.

#### Measurement of knee extension force (KE-F)

The maximal voluntary isometric KE-F of the right leg was determined using a specially designed myometer (VTK-002R/L; Takeikiki, Tokyo, Japan) in accordance with the procedure described in a previous study [5]. In the maximal test, the subjects executed a 2- to 3-s maximal voluntary contraction two times, with at least 1 min of rest between the trials. If the difference between the two values was more than 10 %, the KE-F was measured once more [14]. The highest KE-F value of the 2 or 3 measurements was adopted [14].

#### Sit-to-stand (STS) test

The time (STS time) required to perform a 10-times-repeated STS test on a molded steel chair (0.40 m height and 0.36 m depth) was determined in accordance with the procedure described in a prior study [5]. In the measurements, the subjects were asked to stand up from a sitting position and then to sit down 10 times as fast as possible. The STS time was recorded using a stopwatch to the nearest tenth of a second. The subjects performed the STS test two times with an interval of 1 min between the trials. The faster time was adopted for the individual data. STS power index (STS-PI) was calculated using the following equation, which is a modification of an equation described by Takai et al. [5]: STS-PI = {(Ht − 0.4) × body mass × 10}/STS time, where 0.4 (m) and Ht (m) represent the height of the chair and body height, respectively. In a prior examination using 556 Japanese women aged 50–94 years, the corresponding values calculated by the equation used here were highly correlated with those by the equation developed by Takai et al. [5] (*r* = 0.962). In addition, velocity index in the STS task (STS-VI) was calculated by the equation mentioned above, in which body mass was not included.

To compare among the rates of age-related changes in KE-F, KE-F relative to body mass (KE-F/BM), STS-PI, and STS-VI, we calculated the percentages of their values to the mean value of the corresponding variable for YOG. These values are referred to as %KE-F, %KE-F/BM, %STS-PI, and %STS-VI, respectively. The repeatability of KE-F and STS time measurements has been certified in a prior study [6].

#### Statistics

Descriptive values are presented as means ± SDs. A two-way analysis of variance (ANOVA) with a Scheffe test was used to test the effects of age and sex on the measured variables. If a significant interaction of age and sex was found, Student’s unpaired *t*-test was used to examine the differences between the age groups within the same sex and between men and women in the same age group. A simple linear regression analysis was used to calculate the coefficient of correlation between age and each of %KE-F and %STS-PI. For these relationships, an analysis of covariance (ANCOVA) was used to test the homogeneity of the regression slopes between men and women [15]. As a result of ANCOVA, if the interaction of age and sex was significant, the regression slopes were considered to be significantly different between men and women. In addition, Student’s unpaired *t*-test was used to examine the difference between the men and women of OOG in each of %KE-F, %KE-F/BM, %STS-PI, and %STS-VI. The probability level for statistical significance was set at *p* < 0.05.

## Results

KE-F, KE-F/BM, and STS-PI had significant effects of age (*p* < 0.0001) and sex (*p* < 0.001–*p* < 0.0001) without the interaction of the two factors (Table 2), indicating that these variables were significantly higher in YOG than in OOG (*p* < 0.0001) and in men than in women (*p* < 0.001–*p* < 0.0001). STS time and STS-VI had significant interaction of sex and age (*p* < 0.0001 and *p* < 0.01, respectively). STS time was significantly greater in OOG than in YOG for both sexes (*p* < 0.0001) and in women than in men for OOG (*p* < 0.0001). STS-VI was significantly higher in YOG than in OOG for both sexes (*p* < 0.0001) and in men than in women for the two age groups (*p* < 0.0001).

**Table 2:** Descriptive data on the measured variables

				Two-way ANOVA		
Variables	Sex	YOG	OOG	Age	Sex	Age × sex
KE-F, N	M	307.7 ± 80.0^ab^	263.9 ± 88.2^b^	*p* < 0.0001	*p* < 0.0001	*p* = 0.521
	W	241.1 ± 65.9^a^	205.4 ± 67.7			
KE-F/BM, N/kg	M	4.937 ± 1.247^ac^	4.435 ± 1.392^c^	*p* < 0.0001	*p* < 0.001	*p* = 0.842
	W	4.547 ± 1.233^a^	4.088 ± 1.298			
STS time, s	M	12.84 ± 3.15^d^	15.37 ± 5.15^e^	*p* < 0.0001	*p* < 0.0001	*p* < 0.0001
	W	13.39 ± 3.71^d^	19.60 ± 6.90			
STS-PI	M	63.5 ± 16.2^ab^	51.9 ± 17.5^b^	*p* < 0.0001	*p* < 0.0001	*p* = 0.074
	W	48.0 ± 13.6^a^	31.7 ± 13.1			
STS-VI	M	1.02 ± 0.23^de^	0.87 ± 0.27^e^	*p* < 0.0001	*p* < 0.0001	*p* < 0.01
	W	0.90 ± 0.24^d^	0.62 ± 0.23			

Values are means ± SDs

*M* men, *W* women, *YOG* younger-old group, *OOG* older-old group, *KE-F* knee extension force, *KE-F/BM* KE-F relative to body mass, *STS time* time taken for a 10-times-repeated STS task, *STS-PI* power index calculated by using an equation with body height, body mass, and STS time as variables, *STS-VI* velocity index calculated using an equation with body height and STS time as variables

^a^Significantly different from OOG at *p* < 0.0001 as a result of Scheffe test

^b^Significantly different from women at *p* < 0.0001 as a result of Scheffe test

^c^Significantly different from women at *p* < 0.001 as a result of Scheffe test

^d^Significantly different from OOG at *p* < 0.0001 as a result of unpaired *t*-test within the same sex

^e^Significantly different from women at *p* < 0.0001 as a result of unpaired *t*-test within the same age group

**Fig. 1:**
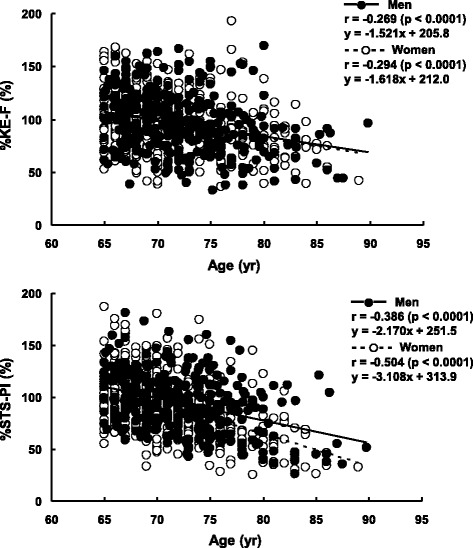
Relationship between age and each of %KE-F (*upper*) and %STS-PI (*lower*)

%KE-F and %STS-PI were negatively correlated to age in both men (*r* = −0.269 and *r* = −0.386, respectively, *p* < 0.0001) and women (*r* = −0.294 and *r* = −0.504, respectively, *p* < 0.0001) (Fig. 1). The regression slope in the relationship between age and %KE-F was −1.521 for men and −1.618 for women, and that in the relationship between age and %STS-PI was −0.217 for men and −3.108 for women. The results of ANCOVA revealed that the regression slopes in the relationship between age and %KE-F were not significantly different between men and women (*p* = 0.808). On the other hand, the corresponding analysis on the relationship between age and %STS-PI showed that the regression slopes significantly differed between men and women (*p* < 0.05). The results of ANCOVA on the relationships between age and either %KE-F or %STS-PI within the same sex indicated that the regression slopes for men were similar between the two regression lines (*p* = 0.104), but those for women were significantly different (*p* < 0.001).

Table 3 shows descriptive data on %KE-F, %KE-F/BM, %STS-PI, and %STS-VI in OOG. %KE-F and %KE-F/BM were similar between men and women, but %STS-PI and %STS-VI were significantly lower in women than in men (*p* < 0.001 and *p* < 0.0001, respectively).

**Table 3:** Descriptive data on %KE-F, %KE-F/BM, %STS-PI, and %STS-VI in older-old group

Variables	Men	Women
%KE-F, %	85.8 ± 28.7	85.2 ± 28.1
%KE-F/BM, %	89.8 ± 28.2	89.9 ± 28.6
%STS-PI, %	81.7 ± 27.5	66.1 ± 27.3^b^
%STS-VI, %	85.6 ± 26.2	69.2 ± 25.4^a^

Values are means ± SDs

*%KE-F* KE-F value relative to the mean value of younger-old group, *%KE-F/BM* KE-F/BM value relative to the mean value of younger-old group, *%STS-PI* STS-PI value relative to the mean value of STS-PI of younger-old group, *%STS-VI* STS-VI value relative to the mean value of STS-VI of younger-old group

^a^Significantly different from men at *p* < 0.0001 as a result of unpaired *t*-test

^b^Significantly different from men at *p* < 0.001 as a result of unpaired *t*-test

## Discussion

The regression slope in the relationship between age and %STS-PI was significantly steeper in women than in men, without the corresponding difference in the relationship between age and %KE-F (Fig. 1). Furthermore, %KE-F and %KE-F/BM were almost the same between men and women of OOG, but %STS-PI was significantly lower in women than in men (Table 3). These results support our hypothesis that, as compared to older men, older women would show a steeper age-related loss in STS-PI, with greater magnitude than that in knee extensor strength.

As described earlier, no studies have tried to clarify whether age-related loss in power development during the whole-body movement such as the STS task differs between elderly men and women, in relation to that in the force generation capability of the prime movers. On the other hand, two studies have already examined the sex difference in the age-related loss of power generation capability during explosive leg extension task [16, 17]. In these studies, the rates of age-related difference in knee extensor strength were similar between men and women, as observed in the current study. However, the previous findings on the corresponding difference in leg extension power differ from report to report. For example, as a result of comparison between subjects aged 20 and 80 years, Samson et al. [16] observed that the relative differences between the two age groups in leg extension power was higher in women (61 %) than in men (49 %). However, Skelton et al. [17], who examined individuals in the same age range (65–89 years) as those in the current study, reported that men lost leg extension power more rapidly than women. The current results disagree with this latter finding.

The previous studies cited above determined leg extension power by using an apparatus specially designed for measuring mechanical power during an explosive movement involving hip and knee extensions in a seated position. STS is a whole-body movement without support. Thus, we cannot directly compare the current and previous findings. To our knowledge, only one study has examined sex difference in power generation capability during STS task on the basis of the difference between younger and older individuals in it [7]. From the limited findings, peak power during STS task was 110 % higher in older men than in older women, whereas the sex difference in young adults was only 22 %. From a previous finding based on the a retrospective analysis of top age-group weightlifting and powerlifting records, the rates of age-related declines in performances were greater in women than in men in the tasks that require greater involvement of explosive power and complex movement [9]. The execution of STS task involves muscle strength development of the lower body and wide ranges of joint motion [2], and it requires the performer to exert muscle strength with appropriate magnitude and timing [8]. Taking these aspects into account, the observed higher rate of age-related loss in STS-PI in women may be assumed to be partially attributable to the nature of the STS task, namely, whole-body movement without support.

We have no data concerning the physiological background for the higher rate of age-related loss in STS-PI for women. As a reason for the current result, sex difference in the effect of aging on muscle function during explosive and rapid movement might be involved. The STS task adopted here required the subjects to repeat sit-to-stand movement ten times with maximal effort. This may induce the prime movers a couple of eccentric and concentric muscle contractions through a series of sitting down and standing up, being a phenomenon similar to stretch-shortening cycle (SSC) as performed in a counter-movement test [18]. From the findings of Caserotti et al. [18], force and power generation levels during the eccentric phase in counter-movement jump were similar between men and women aged 75 years, but the men had higher peak muscle power during the concentric phase than the women. In addition, it has been reported that older women depend more on muscle contraction velocity to perform different types of functional task [19], and deterioration in the maximal contraction velocity is linked to the onset of functional difficulties in older women [20]. The maximum shortening velocities of type I and type IIA fibers have been shown to be slower in older women than in older men, but the corresponding differences are not found in younger subjects [21]. Considering these findings, it seems that the observed sex difference in the age-related loss in %STS-PI might be attributable to greater impairment in muscle function during explosive and rapid movement, notably in the concentric phase, in women.

STS performances in the elderly involve various factors such as sensation, speed, balance, and psychological status in addition to muscle strength [3]. In addition, biomechanical analyses have suggested age-related difference in movement strategies during stand from a chair [22–25]. For example, Gross et al. [22] reported that elderly women generated more trunk flexion and horizontal momentum while still in contact with the chair. Besides the assumption mentioned above, therefore, we cannot rule out that other factors affecting STS performance might be linked to the observed sex difference in the age-related loss of %STS-PI. To our knowledge, however, there is no information on how the existence of sex differences in the factors described here can be associated with those in KE-F and STS-PI.

Furthermore, functional status in the elderly has an influence of habitual physical activity level [26]. A cross-sectional study showed significant age-related loss of the Metabolic Equivalent of Task in total physical activity without an effect of sex on the patterns in activity profiles [27]. However, a longitudinal study [28], in which men and women aged 46 to 80 years were examined on 2 occasions separated by a mean time of 9.4 years, indicated that the levels of sports and recreational activities decreased more in men than in women. If this finding can be applied to the subjects examined here and the age-related change in physical activity level is associated with that in power generation capability, the age-related loss in STS-PI might be assumed to be greater in men than in women. However, the current results differ from this. In any case, the present study did not perform the measurements on the levels of physical activities during daily life in the subjects. With regard to the influences of the physical activity levels on the observed difference in %STS-PI between men and women, further investigation is needed to clarify this.

It is known that, for improving the performance of activities of daily living in older adults, power training that contains high-velocity contractions is more effective than standard resistance training that mainly aims to increase muscle strength [29]. Apart from the reasons for the observed sex difference in age-related loss in STS-PI, the current results suggest a higher priority of improving the power generation capability in daily actions such as STS for older women than for older men. Notably, consideration of the training modality with a focus on enhancing the velocity component of power will be essential because women showed a steeper age-related loss in STS-PI than in KE-F, but the corresponding difference was not found in men.

## Conclusion

The present study indicates that, in Japanese men and women aged 65 years or over, the rate of age-related loss in power production in sit-to-stand task is higher in women than in men, with greater magnitude than that in knee extension force. This suggests a higher priority of improving power generation capability during whole-body movement such as STS in older women than in older men.

## Abbreviations

ANCOVA: analysis of covariance
ANOVA: analysis of variance
KE-F: knee extension force
KE-F/BM: KE-F relative to body mass
M: men
OOG: older-old group
SSC: stretch-shortening cycle
STS test: sit-to-stand test
STS time: time for a 10-times-repeated STS test
STS-PI: power index calculated from the score of a 10-times-repeated STS test
STS-VI: velocity index calculated from the score of a 10-times-repeated STS test
W: women
YOG: younger-old group
%KE-F: KE-F value relative to the mean value of the younger-old group
%KE-F/BM: KE-F/BM value relative to the mean value of the younger-old group
%STS-PI: STS power index value relative to the mean value of the younger-old group
%STS-VI: STS velocity index relative to the mean value of the younger-old group

## References

[1] Smith WN, Del Rossi G, Adams JB, Abderlarahman KZ, Asfour SA, Roos BA (2010). Simple equations to predict concentric lower-body muscle power in older adults using the 30-second chair-rise test: a pilot study. Clin Interv Aging.

[2] Papa E, Cappozzo A (2000). Sit-to-stand motor strategies investigated in able-bodied young and elderly subjects. J Biomech.

[3] Lord SR, Murray SM, Chapman K, Munro B, Tiedemann A (2002). Sit-to-stand performance depends on sensation, speed, balance, and psychological status in addition to strength in older people. J Grontol: Med Sci.

[4] Csuka M, McCarty DJ (1985). Simple method for measurement of lower extremity muscle strength. Am J Med.

[5] Takai Y, Ohta M, Akagi R, Kanehisa H, Kawakami Y, Fukunaga T (2009). Sit-to-stand test evaluate knee extensor muscle size and strength in the elderly: a novel approach. J Physiol Anthropol.

[6] Kanehisa H, Fukunaga T (2014). Age-related change in sit-to-stand power in Japanese women aged 50 years or older. J Physiol Anthropol.

[7] Petrella JK, Kim J, Tuggle SC, Hall SR, Bamman MM (2005). Age differences in knee extension power, contractile velocity, and fatigability. J Appl Physiol.

[8] Linderman U, Muche R, Stuber M, Zijlstra W, Hauer K, Becker C (2007). Coordination of strength exertion during the chair-rise movement in very old people. J Gerontol Med Sci.

[9] Anton MM, Spirduso WW, Tanaka H (2004). Age-related declines in anaerobic muscular performance: weightlifting and powerlifting. Med Sci Sports Exercise.

[10] Bassey EJ, Fiatarone MA, O’neill EF, Kelly M, Evans WJ, Lipsitz LA (1992). Leg extensor power and functional performance in very old men and women. Clin Sci.

[11] Raj IS, Bird SR, Shield AJ (2010). Aging and the force-velocity relationship of muscles. Exp Gerontol.

[12] Butler AA, Menant JC, Tiedemann AC, Lord SR (2009). Age and gender differences in seven tests of functional mobility. J Neuroeng Rehabil.

[13] American College of Sports Medicine. ACSM’s guidelines for exercise testing and prescription (Seventh Edition) (Japanese version). Translation supervised by the Editorial Board of the Journal of Physical Fitness and Sports Medicine in the Japanese Society of Physical Fitness and Sports Medicine, Nankodo, Tokyo, 2006.

[14] Yoshitake Y, Takai Y, Kitamura M, Kawanishi M, Kanehisa H (2011). Body mass-based exercise in middle-aged and older women. Int J Sports Med.

[15] Nindle BC, Mahar MT, Harman EA, Patton JF (1995). Lower and upper body anaerobic performance in male and female adolescent athletes. Med Sci Sports Exerc.

[16] Samson MM, Meeuwsen IB, Crowe A, Dessens JA, Duursma SA, Verhaar HJ (2000). Relationships between physiological performance measures, age, height and body weight in healthy adults. Age Ageing.

[17] Skelton DA, Greig CA, Davies JM, Young A (1994). Strength, power and related functional ability of healthy people aged 65–89 years. Age Ageing.

[18] Caserotti P, Aargaard P, Simonsen EB, Puggaard L (2001). Contraction-specific differences in maximal muscle power during stretch-shortening cycle movements in elderly males and females. Eur J Appl Physiol.

[19] Sayers SP, Guralnik JM, Thombs LA, Fielding RA (2005). Effect of leg muscle contraction velocity on functional performance in older men and women. J Am Geriatr Soc.

[20] Van Roie E, Verschueren SM, Boonen S, Bogaerts A, Kennis E, Coudyzer W (2011). Force-velocity characteristics of the knee extensors: an indication of the risk for physical frailty in elderly women. Arh Phys Med Rehabil.

[21] Krivickas LS, Suh D, Wilkins J, Hughes VA, Roubenoff R, Frontera WR (2001). Age- and gender-related differences in maximum shortening velocity of skeletal muscle fibers. Am J Phys Med Rehabil.

[22] Gross MM, Stevenson PJ, Charette SL, Pyka G, Marcus R (1998). Effect of muscle strength and movement speed on the biomechanics of rising from a chair in healthy elderly and young women. Gait Posture.

[23] Scarborough DM, Krebs DE, Harris BA (1999). Quadriceps muscle strength and dynamic stability in elderly persons. Gait Posture.

[24] Schultz AB, Alexnder NB, Ashton-Miller JA (1992). Biomechanical analysis of rinsing from a chair. J Biomech.

[25] Ikeda ER, Schenkman ML, Rlley PO, Hodge WA (1991). Influence of age on dynamics of rising from a chair. Phys Ther.

[26] Foldvari M, Clark M, Laviolette LC, Bernstein MA, Kaliton D, Castaneda C (2000). Association of muscle power with functional status in community-dwelling elderly women. J Gerontol: Med Sci.

[27] Milanovic Z, Pantelic S, Trajkovic N, Sporis G, Kostic R, James N (2013). Age-related decrease in physical activity and functional fitness among elderly men and women. Clin Interv Aging.

[28] Hughes VA, Frontera WR, Roubenoff R, Evans WJ, Singh MA (2002). Longitudinal changes in body composition in older men and women: role of body weight change and physical activity. Am J Clin Nutr.

[29] Hazel T, Kenji K, Jakobi J (2007). Functional benefit of power training for older adults. J Aging Phys Act.

